# Self-poisoning with pesticides in Jiangsu Province, China: a cross-sectional study on 24,602 subjects

**DOI:** 10.1186/s12888-020-02882-9

**Published:** 2020-11-23

**Authors:** Boshen Wang, Lei Han, Jinbo Wen, Juan Zhang, Baoli Zhu

**Affiliations:** 1 Key Laboratory of Environmental Medicine Engineering of Ministry of Education, School of Public Health, Southeast University, Nanjing, 210009 Jiangsu China; 2 Institute of Occupational Disease Prevention, Jiangsu Provincial Center for Disease Prevention and Control, No. 172 Jiangsu Road, Nanjing, Jiangsu China; 3 Department of Chronic Disease Prevention and Control, Huai’an City Center for Disease Control and Prevention, Huai’an, 223001 China

**Keywords:** Self-poisoning, Pesticide, Cross-sectional study

## Abstract

**Background:**

With an estimated > 800,000 suicide-related deaths and potentially several attempts for each death in the world. The purpose of this study was to determine the epidemiological characteristics of self-poisoning with pesticides within the Jiangsu province in China.

**Methods:**

Suicide rate was calculated the Routine Surveillance System by Jiangsu Provincial Center for Disease Control and Prevention, stratified by gender, age and region, combined with socioeconomic and agriculture-related factors to investigate trends in suicide over the study period. A logistic regression model was used to investigate the associations between pesticide types and pesticide-related deaths.

**Results:**

In recent years, Jiangsu Province has witnessed a decrease in pesticide self-poisoning cases and consequent deaths. Among all suicides by deliberate ingestion of pesticides, the proportion of cases were mainly in the age 40, accounting for 3.43% of all cases with pesticide suicide. The proportion of suicide due to pesticide poisoning in females was markedly higher than that in males (*p* < 0.001). Suicide using organophosphate and carbamate insecticides was most common, with 10,303 reported cases accounting for 42.02% of all suicides.

**Conclusions:**

For national responses to be effective, the characteristics of pesticide suicides should be comprehensively investigated for the formulation of corresponding prevention strategies. At present, more pesticide suicide prevention policies for the elderly people and women should be implemented, and stronger pesticide management policies should be implemented for rural areas.

## Background

Suicide is a universal incident, with an estimated 800,000 suicide-related deaths reported worldwide in 2016. On average, the annual worldwide suicide rate is 10.5 per 100,000 individuals [1]. Each suicide has an extreme negative impact on close relatives and society on a global scale. Developing countries have a high suicide rate of 79% [2]. The percentage of suicides due to pesticide self-poisoning differs widely among regions, from 0.9% in Europe to 48.3% in Western Pacific countries [3]. Among the low- and middle- income countries [4], the suicide rate through pesticide self-poisoning is high in agricultural areas, contributing to 30% of the world suicide rate. The overall suicide rate in Asia is approximately 19.3 per 100,000, about 30% higher than the global rate of 16.0 per 100,000 [5]. WHO has announced a Global Pesticides and Health Initiative in view of pesticide ingestion being the most common method of suicide worldwide [6].

Suicides in China account for 26% of the global suicide statistics. Suicide is the fifth leading cause of death in the country, along with injuries, poisoning and falls. China is an agricultural and developing nation with a large number of rural inhabitants. In rural areas, higher rates of suicide are predicted relative to urban areas [7]. Intensive usage of pesticides (~ 1.4 million tons annually) has been reported in China [8]. Notably, suicide by pesticide poisoning is strongly associated with the proportion of the pesticide sold and management policies [9]. The most common method of suicide in China was pesticide poisoning. Although the proportion of suicides using pesticides in China has decreased from approximately 62% [10] to 49% [11], self-poisoning with pesticides is still the leading cause of suicide. Furthermore, the western models in which suicide is considered the direct result of mental illness, while the Western model that focuses most of the prevention efforts on the identification and treatment of mental illness may not be applicable to China [12, 13]. Suicide in China, especially pesticide suicide, is mainly impulsive suicide, and the main influencing factors are marital and family conflicts [14].

Suicide is a serious public health problem but preventable with timely, evidence-based, and often low-cost interventions. For national responses to be effective, a comprehensive multisectoral suicide prevention strategy is warranted. From this viewpoint, we investigated the characteristics of 24,602 pesticide-based suicides in China’s Jiangsu Province over the past 13 years in the current study. Our findings offer a scientific basis for developing effective intervention measures as well as preventive strategies.

## Methods

### Data sources

The reporting system database contained information obtained from health institutions, hospitals and community healthcare centres. Pesticide poisoning data were acquired through the routine surveillance system of Jiangsu CDC Usage of the database was approved by CDC of Jiangsu. Jiangsu is one of the richest agricultural provinces in China and has the highest GDP per capita of Chinese provinces and second-highest GDP of Chinese provinces, after Guangdong. The Engel coefficient is the proportion of family income that is spent on food [15]. Engels’ law is an economic observation that as income rises, the proportion of income spent on food falls-even if absolute expenditure on food rises. In other words, the income elasticity of food demand is between 0 and 1. It is a reflection of a country’s standard of living. As the proportion (the Engel coefficient) increases, the country becomes poorer. In contrast, a lower Engel’s coefficient indicates a higher standard of living. Socioeconomic (% Engel coefficient, Unemployment rate, Divorce rate, GDP per capita (US$)) and agriculture-related (% population in farming, Pesticide sold (10,000 ton)) data were from the National Bureau of Statistics.

### Quantity and characteristics of the pesticides sold and used

China is an agricultural and developing nation with a large number of rural inhabitants. After the announcement of No. 322 of the Ministry of Agriculture at the end of 2003, the use of highly toxic pesticides in Jiangsu Province showed an obvious downward trend. By 2007, five highly toxic pesticides such as methamidophos and parathion-methyl were basically stopped selling. The amount of pesticide sold decreased at an average rate of 2% per year from 2006 to 2018 (Fig. 6 A). According to the preliminary analysis of the plant protection and quarantine station of Jiangsu Province, insecticides accounts for 30–45%, fungicide accounts for 25–40%, herbicide accounts for 20–30% and rodenticide accounts for 0.2–1%.

### Characteristics of included cases

All participants (24,602 cases) were diagnosed by specialists at different levels in the hospital according to the correlated national diagnostic criteria. The pesticide poisoning report cards contained vital information, such as patient age, sex, region and other diagnostic results. Among the poisoning records obtained through the ICD-10 code (T36.0-T65.9), all records from January 1, 2006 to December 31, 2018, listed a disease diagnostic code for at least one pesticide toxicity effect (ICD-10 code, T60.0-T60.9). For our study, all doctor specialists were briefly trained on judging and reporting suicide. The information of suicidal deaths gathered at the county CDCs were then forwarded on a monthly basis to the provincial CDC. For those suicidal deaths that were not recognized by any health agency, our mortality registry system allowed the village treasurers, who collect fees for each burial or cremation and are aware of all the deaths in the village, to notify the township health agency or the county CDC. Pesticide deaths included those who died before admission and those who failed treatment after admission.

### Analysis of data

The total population of Jiangsu Province from 2006 to 2018 was 78.69–80.5 million. Rural population of Jiangsu Province from 2006 to 2017 was 36.82–25.08 million. The incidence of pesticide self-poisoning was calculated per 1,000,000 population. Detailed statistical analyses on the sociodemographic variables of pesticide self-poisoning were conducted. Data were compiled in the statistical package for social sciences (SPSS) software, version 20.0, and analyzed by application of Mann-Whitney U or Student’s *t*-test for continuous data. Based on the significance level of equality of variance, one-way ANOVA or Welch F test was applied to compare mean values. Heat maps showing correlation between pesticide suicides and socioeconomic and agriculture-related factors were generated by Heml 1.0 (hemi.biocuckoo.org). We used logistic regression model to analyze the association between pesticide suicide deaths (vs no deaths) and pesticide types. The dependent variable was pesticide suicide deaths (vs no deaths). Confounding factors controlled in the multivariate analysis are age, sex, and different types of pesticides. Logistic regression models are presented as odds ratios (OR) and 95% confidence intervals (CI). *P* values < 0.05 were considered statistically significant.

## Results

**Fig. 1:**
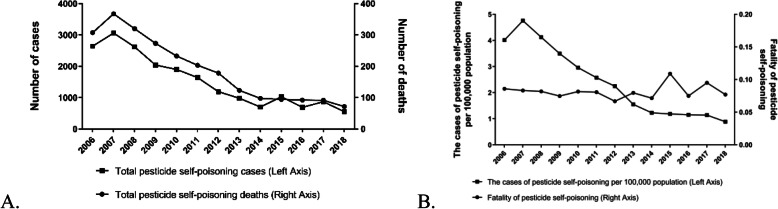
**a** Distribution of cases and deaths based on pesticide poisoning by year. **b** Fatality of pesticide self-poisoning and proportion of cases per 100,000 population by year

From 2006 to 2018, a total of 24,602 cases of self-poisoning through pesticides were reported in Jiangsu Province. In 1991 cases, the individuals died, leading to a fatality rate of 8.09%. We observed a general decline in the number of self-poisoning cases and deaths with pesticides, in particular, from 2007 to 2014 (Fig. 1a). However, despite the decreasing trend in the proportion of pesticide self-poisoning cases per million population (Fig. 1b), case fatality rates were not significantly different in Jiangsu from 2006 to 2018 (*P* > 0.05).

**Fig. 2:**
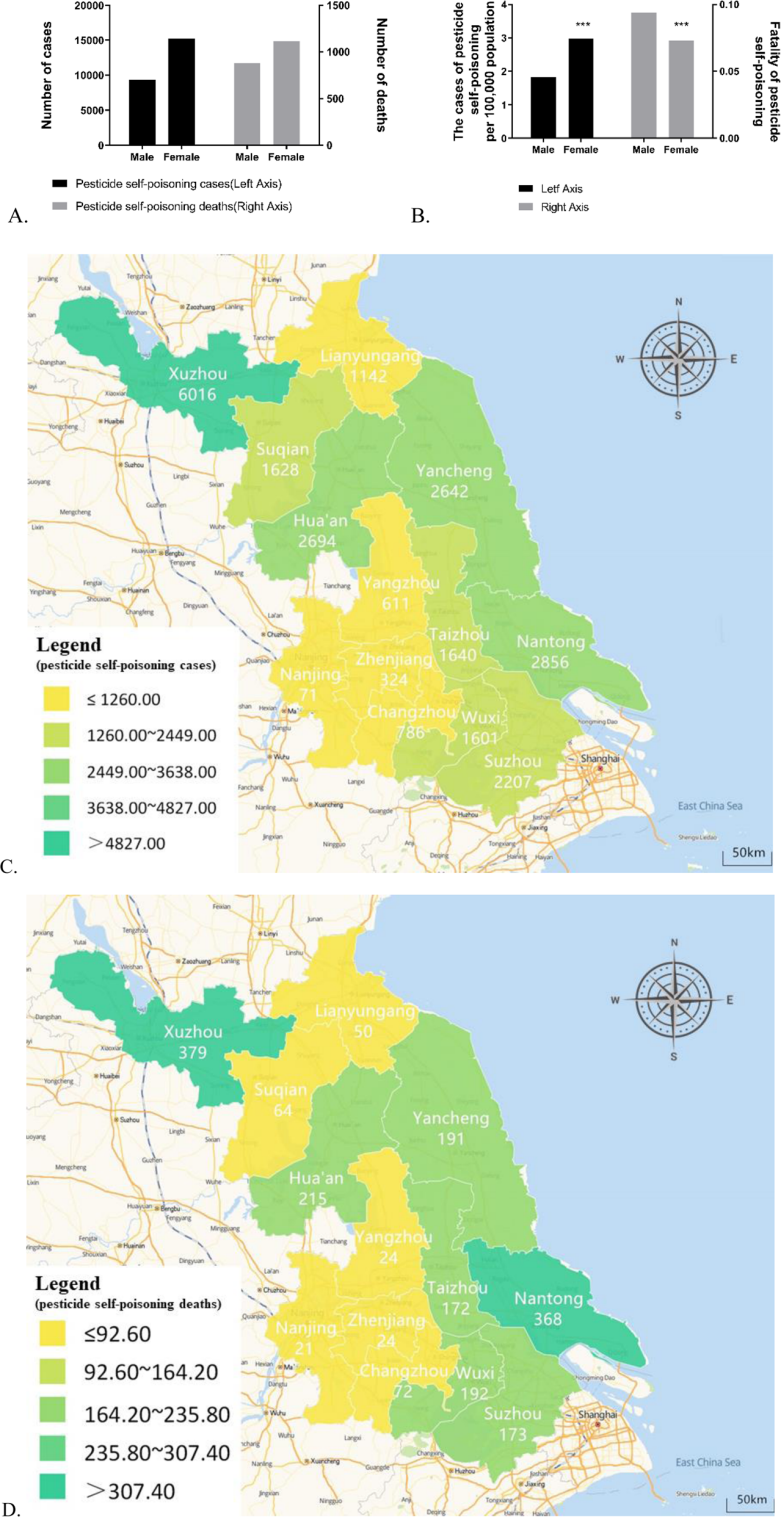
**a** Distribution of pesticide poisoning cases and deaths by sex. **b** Fatality and proportion of pesticide self-poisoning cases by sex per 100,000 population (***P < 0.001 relative to pesticide self-poisoning cases among males). **c-d** Distribution of pesticide self-poisoning cases and deaths in Jiangsu Province based on city polygons (2006–2018). (The map depicted in Fig. 2 was designed by our own team)

In total, 9353 male and 15,249 female suicides by intentional ingestion of pesticides were recorded. The proportion of pesticide-induced suicides in females (~ 2.97 per 100,000 individuals sex-adjusted within the Jiangsu province population) was significantly higher than that in males (~ 1.82 per 100,000 people) (OR = 0.61, 95% CI: 0.60–0.63, *p* < 0.001), as illustrated in Fig. 2a-b. However, the case fatality rate in female patients was markedly lower than that in male patients (OR = 1.32, 95% CI: 1.20–1.45, *p* < 0.001). From a geographical viewpoint, the proportion (%) of self-poisoning with pesticides in the northern areas of Jiangsu (including Huai’an, Yancheng, Suqian, Lianyungang and Xuzhou) were as high as 58.01, 21.16% in the middle areas (including Nantong, Taizhou, and Yangzhou) and 20.83% in the southern areas (including Changzhou, Suzhou, Nanjing, Wuxi, Zhenjiang and Suzhou), as shown in Fig. 2c. Among these, the top three cities with the highest number of cases of pesticide self-poisoning were City of Xuzhou (6016, ~ 5.28 per 100,000 population), City of Huai’an (2694, ~ 4.22 per 100,000 population) and City of Nantong (2856, ~ 3.01 per 100,000 population). The highest proportion of attempted suicides with pesticides and consequent deaths occurred in Xuzhou (24.84 and 19.34%, respectively) relative to other regions (Fig. 2d).

**Fig. 3:**
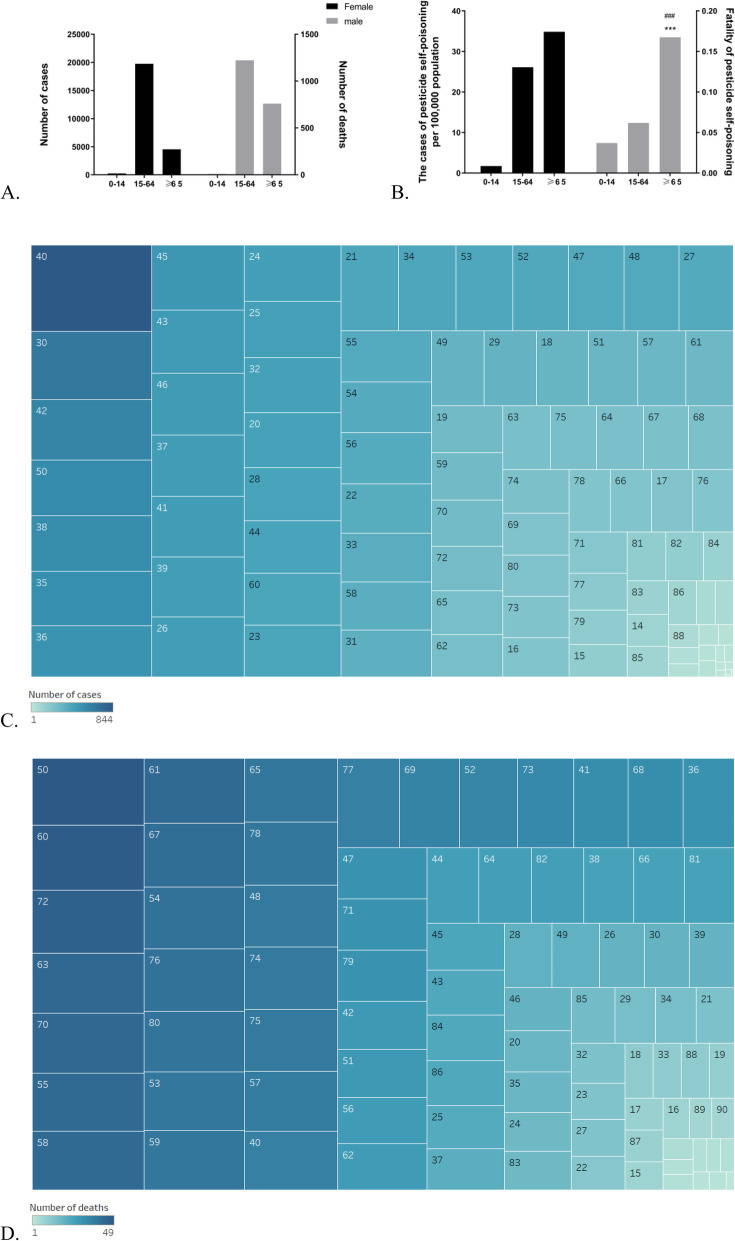
**a** Distribution of pesticide poisoning cases and deaths by sex. **b** Fatality and proportion of pesticide self-poisoning cases by sex per 100,000 population. (*** *P* < 0.001 relative to the 0–14 years age group; ### *P* < 0.001 relative to the 15–64 year age group). **c-d** Heatmap based on distribution of pesticide poisoning cases and deaths by age

A large proportion of pesticide self-poisoning cases (80.23%) was identifiable in the age range of 15–64 years (Fig. 3a). Among all suicides by deliberate ingestion of pesticides, the reported cases were mainly in the age 40, accounting for 3.43% of all cases with pesticide suicide (Fig. 3c). Thus, the proportion of pesticide self-poisoning cases per 100,000 population in the age range of ≥65 was found to be significantly higher than that in the age range of 15–64 (*p* < 0.001) as shown in Fig. 3b. Total pesticide self-poisoning deaths were mainly in the age 50, accounting for 2.46%. Subjects aged above 65 years accounted for higher case fatality rate (*p* < 0.001).

**Fig. 4:**
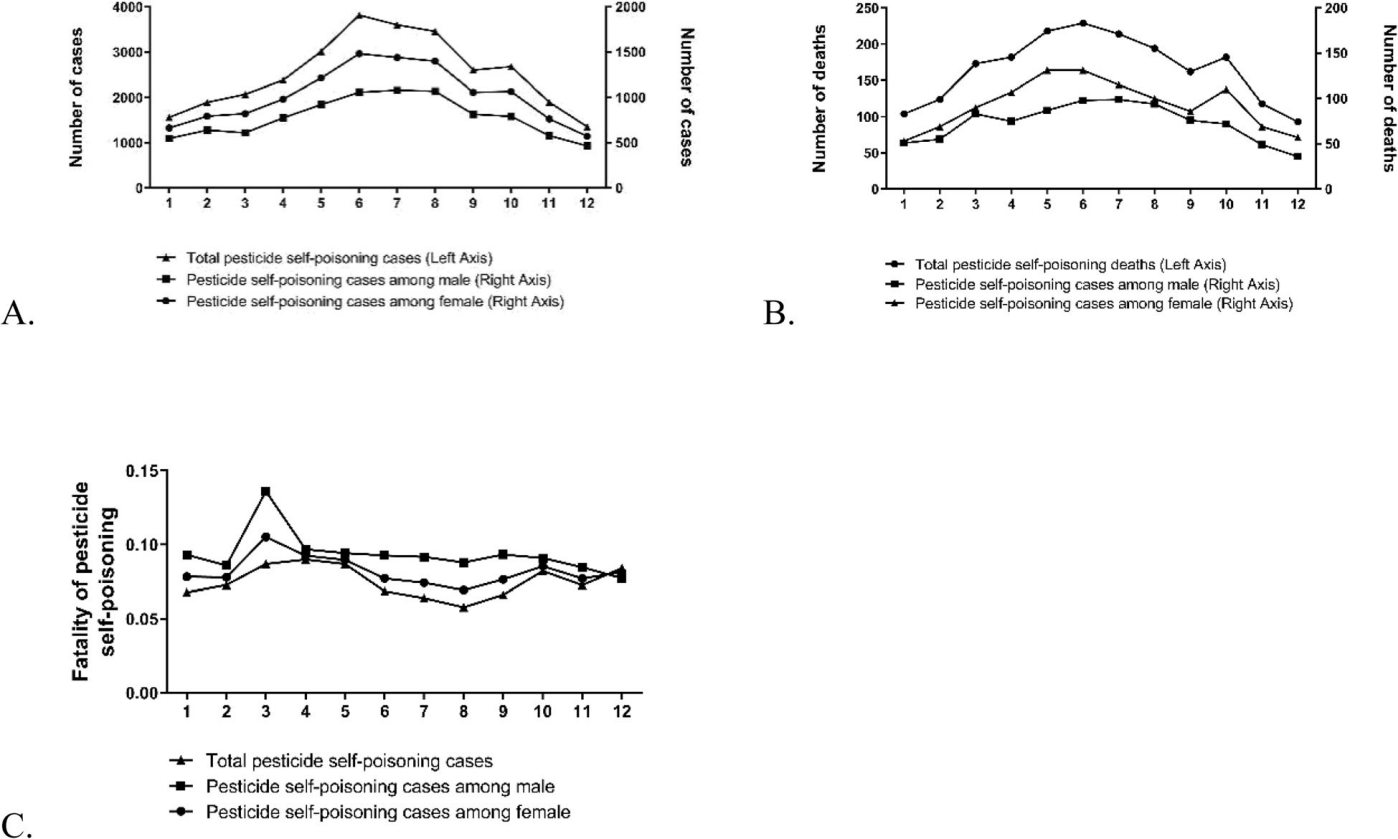
**a-b** Distribution of pesticide self-poisoning cases and deaths by month. **c** Fatality of pesticide self-poisoning cases by month

The patterns of pesticide self-poisoning showed apparent seasonality. Figure 4 presents the distribution of cases and deaths by month and season in both male and female groups. In terms of distribution according to month, pesticide self-poisoning was most common in June (2966 cases) and least common in December (1144 cases; Fig. 4a). Based on season, the majority of reported cases were in the summer (7360, 30.0%) and fall (7793, 31.77%) during the farming season. Male and female groups showed similar distribution of monthly and seasonal pesticide poisoning. The number of cases involving females outnumbered those involving males during all seasons. The monthly pesticide-induced deaths ranged from 93 to 229, with the highest number recorded in June and lowest in December (Fig. 4b). The majority of deaths in males occurred in June while female deaths were mainly reported over May and June.

**Fig. 5:**
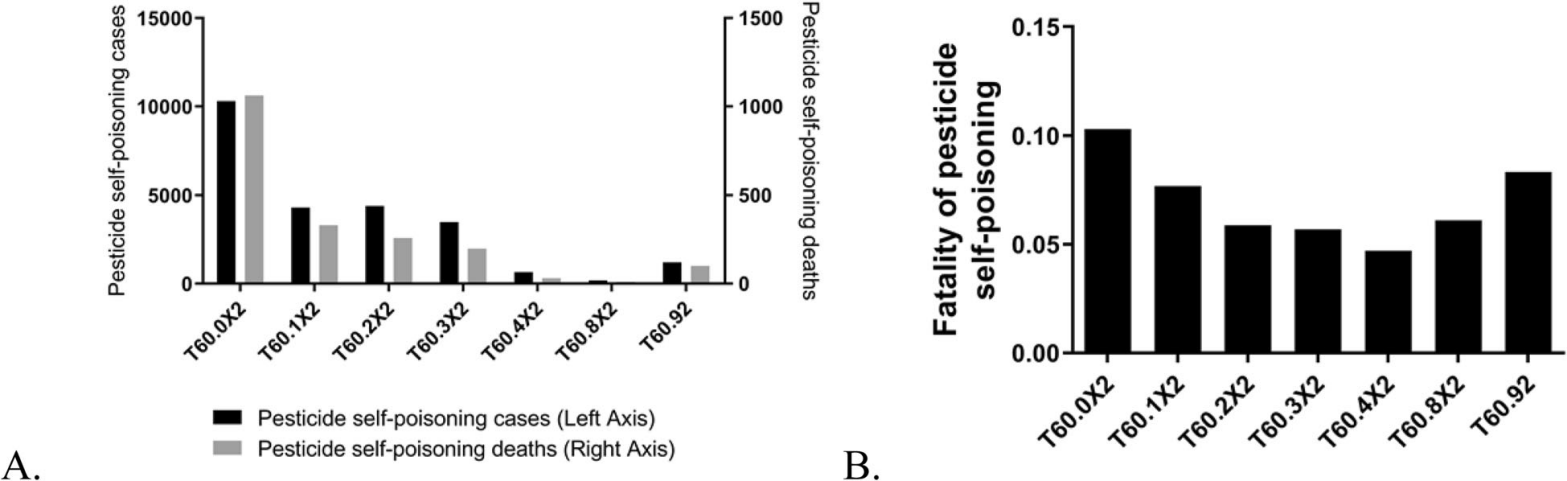
**a-b** Cases of self-poisoning with different types of pesticides

Suicides via poisoning with carbamate and organophosphate insecticides were wide-ranging, totalling 10,303 cases that accounted for 42.02% of all suicides (Fig. 5). The majority of pesticide self-poisoning was attributable to insecticides (19,002; 77.50%). The case-fatality rate induced by ingestion of organophosphate and carbamate insecticides was significantly higher than those using other pesticides (*p* < 0.001). Logistic regression analysis was employed to assess the association between pesticide-related deaths and different types of pesticides to a more precise extent, additionally adjusting for confounding factors (Table 1). Independent variables integrated the age in addition to sex. In terms of the attuned odds ratio of pesticide-induced suicides relative to the “no death” group, subjects with T60.92 exhibited significantly higher risk with OR (95% CI) of 1.143 (1.002–1.304). Compared with organophosphorus pesticide poisoning, which was the unspecified pesticides highest case fatality rate, the OR values were higher after adjusting for age and sex.

**Table 1:** Binary logistic regression analysis of pesticide-related deaths and different types of pesticides adjusted for age, sex and type of pesticide (OR: odds ratio; CI: confidence interval)

Self-poisoning with pesticides		*OR (95% CI)*	*P*
Death (vs No Death)	Age	1.033 (1.031–1.036)	< 0.001
	Sex		
	Male		
	Female	0.862 (0.784–0.948)	0.002
	Different types of pesticides^a^		
	T60.0X2		
	T60.1X2	0.657 (0.553–0.779)	< 0.001
	T60.2X2	0.684 (0.569–0.823)	< 0.001
	T60.3X2	0.499 (0.340–0.731)	< 0.001
	T60.4X2	0.622 (0.332–1.165)	0.138
	T60.8X2	0.937 (0.739–1.189)	0.592
	T60.92	**1.143 (1.002–1.304)**	**0.046**

^a^ T60.0X2: Toxic effect of organophosphate and carbamate insecticides, intentional self-harm

T60.1X2: Toxic effect of halogenated insecticides, intentional self-harm

T60.2X2: Toxic effect of other insecticides, intentional self-harm

T60.3X2: Toxic effect of herbicides and fungicides, intentional self-harm

T60.4X2: Toxic effect of rodenticides, intentional self-harm

T60.8X2: Toxic effect of other pesticides, intentional self-harm

T60.92: Toxic effect of unspecified pesticide, intentional self-harm

**Fig. 6:**
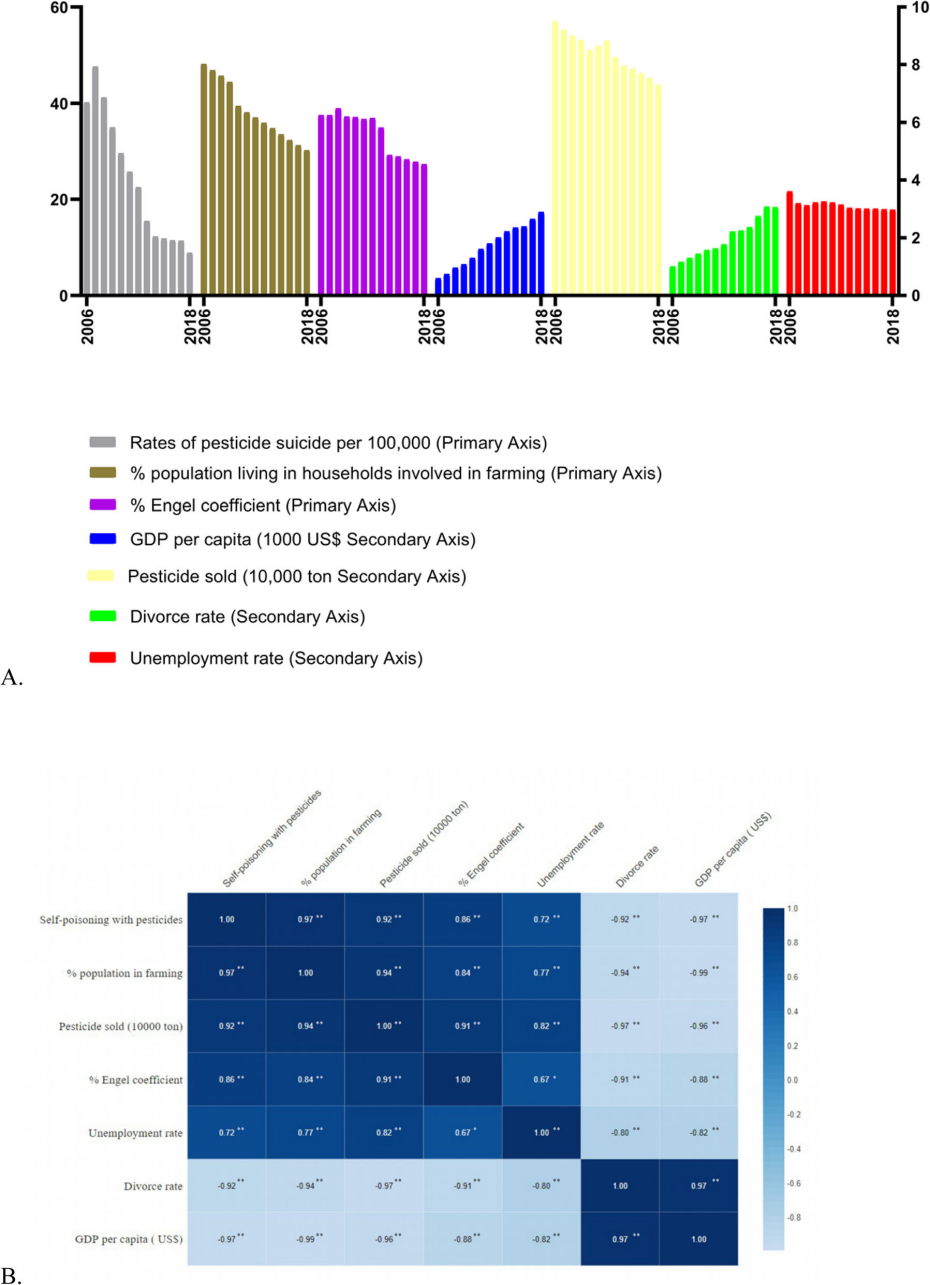
**a** Trends in rate of pesticide suicides and socioeconomic and agriculture-related factors in Jiangsu from 2006 to 2018. **b** Correlation plot showing the heatmap with the Pearson’s correlation coefficient values for Self-poisoning with pesticides, % population in farming, Pesticide sold (10,000 ton), % Engel coefficient, Unemployment rate, Divorce rate, GDP per capita (US$). Color key of the heatmap is shown at the right of the plot. For B data is presented as Pearson’s correlation coefficient values, *p-value< 0.05, **p-value< 0.01

The proportion of the population living in farming households markedly declined over the study period. The unemployment rate steadily decreased during the study period. Divorce rates increased markedly from 2006 to 2018, with 25.41% increase in 2012. GDP per capita markedly increased at an average rate of 14% per year (Fig. 6a). The heat map shows that % population in farming, Pesticide sold (10,000 ton), % Engel coefficient, Unemployment rate, Divorce rate, GDP per capita (US$) and Self-poisoning with pesticides have strong correlation (Fig. 6b).

## Discussion

In the current study, we investigated pesticide self-poisoning cases and consequent deaths in Jiangsu, China, over a 13-year interval. Our data indicated that while Jiangsu province has witnessed a decreasing trend in self-poisoning with pesticides from 2006 to 2018 (*p* < 0.05). Since 2007, the GDP per capita, Divorce rate had increased and % population in farming, Pesticide sold (10,000 ton), % Engel coefficient, Unemployment rate had decreased, and the number of pesticide suicides has dropped by 76.77%. But since 2014, the number of suicides has declined steadily, so we need to find a new key point to reduce the self-poisoning with pesticides.

Globally, the male suicide rate is three times higher than that of females [16, 17]. However, this high male-to-female ratio is primarily a phenomenon in high-income countries, with the 2012 ratio of age-standardized suicide rates being reported as 3.5. In Lower Middle Income Countries the male-to-female ratio is a significantly lower at 1.6, indicating that the proportion of suicide is 57% higher in men than in women [2]. In the current study, the proportion of pesticide self-poisoning in females was significantly higher than that in males (*p* < 0.001). The higher number of self-poisoning cases in females may be due to low intent suicidal behaviour [12]. Self-poisoning with pesticides, especially among women, may be used to gain attention, express distress or get revenge, and not necessarily to end life [18, 19]. Traditionally, suicide is an influential method for people (particularly women), in particular, those with low status, to prove their innocence or protest against unfair treatment [20]. The proportion (%) of self-poisoning with pesticide cases and related deaths were mainly clustered in Northern Jiangsu, accounting for 58.01% of patients in the Province of Jiangsu. In rural areas of China, acquisition of pesticides is relatively easy. Agricultural practices in developed and developing nations are significantly different as less labour is used for cultivation of land in developed nations [21]. Consequently, pesticides are not easily obtainable as they are only available to individuals involved in farming practices while in the less developed regions, pesticides are easily available near homes. Interventions to limit access in such settings are complex and require the involvement of most rural adults, rather than a select few.

According to age, the proportion of suicides were highest in people aged 65 years or older for both men and women worldwide and lowest for people aged less than 15 years. However, age and sex patterns also changed from one region to another [2]. In some regions, the proportion of suicides increased steadily with age while in others, it peaked in young adults and subside in middle age [22]. Notably, suicide cases at 40 years of age were highly reported. Suicide in adults aged 30–49 years accounted for 4.1% of all deaths and represented the fifth major cause of death in China. In addition, incidents of death due to pesticide poisoning increased stepwise with age. China has a different pattern of suicides, with the highest rates reported within the elderly population, compared to other countries [23]. Poor health, increased number of comorbidities, high susceptibility to pesticide poisoning and post-poisoning complications are contributory factors to poor prognosis of the elderly population in China [24].

Previous research has shown that the incidence of self-poisoning with pesticides quarterly in a year due to season-specific agricultural activities, with high the proportion of suicides correlating with availability of pesticides during the farming season [8, 25]. Consistent with earlier findings, we observed higher the proportion of self-poisoning with pesticides in the farming season. Interestingly, the case-fatality rate due to pesticide self-poisoning were highest in March but decreased during the farming season. This was mainly related to the causes of suicide in China. The most striking and puzzling difference between Chinese suicide cases and those from Western countries is the relative importance of mood disorders and other psychopathological conditions as determinants of suicidal behaviour. Mental illness is virtually omnipresent in western literature as a serious risk for suicide but appears to play a less relevant role in Asia, especially China and India [10, 25, 26]. This finding implied that in at least one third of all global suicides, psychiatric disorders do not represent the most relevant risk factor [7].

Our study showed that suicide induction with organophosphate and carbamate insecticides was the most common with a total of 10,303 cases, which accounted for 42.02% of all cases. Overall, insecticides were the main agents utilized for self-poisoning (19,002, 77.50%). The case-fatality rate using organophosphate and carbamate insecticides was significantly higher than those with other pesticides (*p* < 0.001). Organophosphate and carbamate insecticides, such as T60.0X2, were the most commonly used agents, which could be attributed to their prevalent domestic use for controlling mosquitoes and other insects [8]. Although organophosphates and carbamates are most commonly used for agricultural purposes, pyrethroid and other insecticides, such as allethrin, cypermethrin and permethrin, are more frequently used for public health in China. About 73.6% suicides used pesticides stored in the home, mainly insecticides with medium or high toxicity [14]. Studies from other countries have revealed a strong relationship between agent availability and method selected to commit suicide [27]. Method availability may also affect the threshold at which negative life events precipitate suicidal behaviour [28]. This could present further evidence of the relationship between the common use of pesticides for self-poisoning in rural areas and higher the proportion of suicides in China.

With the increase of GDP per capita, the number of suicides had decreased, showing that people’s suicide had a strong negative correlation with per capita GDP. That is, increasing per capita GDP was a key point to reduce pesticide suicide. In addition, the strong correlation between the % population living in households involved in farming and pesticide suicide indicated that pesticide supervision was still weak in agricultural cultivation areas, and it was necessary to improve supervision. Furthermore, there was a strong positive correlation between the decline in cases of pesticide suicide and the decline in pesticide sold, especially since the ban on highly toxic pesticides in 2007, the number of deaths from pesticide suicides had decreased significantly. In addition, there is a weak correlation between the unemployment rate and self-poisoning with pesticides, which coincides with the fact that the main population of pesticide suicides in China is still concentrated in rural area. The unemployed in China are mainly concentrated in urban areas [29]. This phenomenon is also consistent with many other agricultural countries [30, 31], where the suicide rate with pesticide of rural areas is higher.

Suicide prevention strategies focusing on mental disorders may not be the most effective approach for rural China. The government should formulate appropriate policies for the actual situation of local pesticide usage for suicides. First, mental health education in areas with large agricultural populations, such as northern Jiangsu, should be improved. Second, better care for the elderly, who present with the highest the proportion of pesticide suicide, is an essential step. Third, the most important and effective strategy is to increase the management of pesticides and community interventions during the farming season, as well as to screen impulse-related personality, which should effectively reduce the number of impulsive suicide attempts. Finally, there was a need to strengthen the management of pesticides, because the second largest the proportion of pesticide suicide was mainly caused by unspecified pesticides.

Limitations of the study include the limited geographical scope of the sample, which is mainly concentrated in Jiangsu Province, China. Therefore, we are not able to represent the overall situation of pesticide suicide in China and the factors affecting pesticide suicide. Besides, since some pesticide suicide surveys were conducted after the suicide, it is not clear whether the types of pesticides used in the surveys are the same as the suicide. This may also cause recall bias.

## Conclusion

Suicides can be avoided if crucial measures are taken to avert them. For national responses to be effective, the characteristics of pesticide suicides should be comprehensively investigated for the formulation of corresponding prevention strategies. Although the self-poisoning with pesticides cases and deaths has been decreasing year by year, the elderly and women have always been the main groups of suicides. At present, more pesticide suicide prevention policies for the elderly people and women should be implemented, and stronger pesticide management policies should be implemented for rural areas.

## Data Availability

Specific data sets used and/or analysed during the current study are available from the corresponding author on reasonable request.

## Abbreviations

WHO: World Health Organization
CDC: Centers for Disease Control
GDP: Gross Domestic Product
ICD: International Classification of Diseases
ANOVA: One-way analysis of variance
OR: Odds ratios
CI: Confidence intervals
